# Effectiveness of Online Food-Safety Educational Programs: A Systematic Review, Random-Effects Meta-Analysis, and Thematic Synthesis

**DOI:** 10.3390/foods13050794

**Published:** 2024-03-04

**Authors:** Zachary Berglund, Senay Simsek, Yaohua Feng

**Affiliations:** Department of Food Science, Purdue University, 745 Agriculture Mall Drive, West Lafayette, IN 47907, USA; zberglun@purdue.edu (Z.B.); ssimsek@purdue.edu (S.S.)

**Keywords:** systematic review, virtual food-safety education, food safety education, meta-analysis, attitude, knowledge, practices, behavior, food safety, hygiene

## Abstract

Online food-safety educational programs are increasingly important to educate different populations as technology and culture shift to using more technology. However, the broad effectiveness of these programs has yet to be examined. A systematic review, random-effects meta-analysis, and thematic synthesis are conducted to identify the effect size of online food-safety educational programs on knowledge, attitudes, and practices of consumers, food workers, and students and their respective barriers and recommendations. Online food-safety education was found to be of moderate and low effectiveness, with attitudes being the lowest in all populations. Consumers struggled with staying focused, and it was found that messaging should focus on risk communication. Students struggled with social isolation and a lack of time, and it was recommended that videos be used. Food workers struggled with a lack of time for training and difficulty understanding the material, and future programs are recommended to implement shorter but more frequent trainings with simple language. Future online food-safety educational programs should focus on incorporating social elements, as they can remain a huge barrier to learning. They should also focus on changing the participant’s attitude to risk perception and beliefs in the importance of food safety.

## 1. Introduction

Each year, numerous foodborne illnesses are attributed to mishandling or unsafe food handling behaviors [1,2,3]. In the U.S., more than 800 instances of foodborne illness outbreaks are reported annually, and restaurants are implicated in over half of them. When carefully designed and delivered, food-safety educational programs can improve knowledge and change the food preparation behaviors of food handlers [4,5,6]. Prior to the COVID-19 pandemic, food safety educational programs were typically delivered in-person; however, currently, more and more online food-safety educational programs are being developed and delivered [7,8,9,10].

Knowledge about the effectiveness of food safety educational programs is constantly evolving. Young et al. [6] found, with a meta-analysis, that food safety educational programs are effective at increasing the knowledge and inspection scores of various food handlers. Furthermore, tools and technologies have enabled the evolution of online educational programs, a form of asynchronous (classes run on a more relaxed schedule) or synchronous (conducted in real-time) program that uses digital tools to deliver educational instruction without requiring the physical presence of the instructor. Previous literature reviews suggested that online educational programs can be as effective as in-person programs for most work-related topics, including food safety educational topics [11,12].

The effectiveness of online educational programs can vary in different subpopulations. Some of the previous meta-analysis literature containing non-food-related topics suggested that education levels, age, and gender can significantly influence the effectiveness of online educational programs [13,14]. Different populations have different needs, and tailoring educational programs to meet those needs is essential in promoting behavior change [15].

Little is known about the needs of different subpopulations and the effectiveness of online food-safety educational programs in different subpopulations. To address this gap of unknown needs and possible varying effectiveness, a systematic review, meta-analysis, and thematic synthesis was conducted to evaluate the effectiveness of online food-safety educational programs for various food handlers and identify the areas for improving future online food-safety educational programs.

## 2. Materials and Methods

This article is reported following Preferred Reporting Items for Systematic Reviews and Meta-Analyses guidelines (PRISMA) (Document S1) and Enhancing Transparency in Reporting the Synthesis of Qualitative Research (ENTREQ) guidelines [16,17].

### 2.1. Systematic Review

#### 2.1.1. Review Question, Approach, and Eligibility Criteria

This review was developed and adapted to the systematic review design from Young and Thaivalappil [18], Young et al. [19], and Young et al. [6]. The research questions were as follows: (1) What is the effectiveness of online food-safety educational programs among different food handler subpopulations? (2) What is needed to improve the effectiveness of those programs for these subpopulations?

The Population, Intervention, Comparison, Outcome (PICO) framework was used to define the review scope eligibility criteria in Table 1 [20,21]. The populations of interest were students, food workers, and consumers. Students were defined as participants who attended a university or school classroom at the time of the study, which were primarily K-12 and college-level students. Studies focusing on youth under the age of 18 were also assumed to be K-12 students. Food workers were defined as participants in a study aimed at food workers, managers, or other individuals who handle food or train others to handle food as part of their occupation. Consumers were defined as participants in a study targeted to the public who prepare or handle food for household consumption. The outcomes of interest were measures of knowledge, attitudes, and practices (KAP) following an online food-safety educational program. The types of interventions were training courses, workshops, educational messaging materials (e.g., emails and websites), and other theory-based or motivational interventions, so long as they were using digital media with a trainer who was not physically present. Both asynchronous digital educational programs and synchronous virtual web-based workshops were included.

Studies that compared online food-safety educational programs with traditional in-person food safety programs were excluded from this review if they did not contain a defined pre-intervention score or negative control group. The following measures were considered valid for analysis if they measured a component of KAP: (1) inspection scores, (2) coded observed practices, (3) self-reported practices and survey values, and (4) test scores. Relevant study designs included any experimental study with an independent control group, including randomized, controlled trials (RCT), and non-randomized, controlled trials (NCT). Uncontrolled before and after studies (pre-post) (i.e., single group pre-test and post-test comparisons) were also included due to the low availability of studies. Eligible sources of evidence were only peer-reviewed journal articles published in English.

#### 2.1.2. Search Strategy

PICO was used to design keywords for the search strategy, as seen in Table 2. Following the initial keyword search, an additional reverse reference search was conducted by adopting the method from Young et al. to ensure no relevant articles were missed [22].

The titles and abstracts of all search results were screened before assessing acceptance into the analysis by using a pre-specified form containing two questions to determine whether the study met the eligibility criteria and to save a copy of a PDF of the full article. The following questions were included: (1) Does the study discuss an evaluation of an online food-safety educational program for consumers, students, or food workers? (2) Is the study a peer-reviewed journal article or literature review? Studies that answered both questions in the affirmative were saved in Mendeley’s (Elsevier, Amsterdam, The Netherlands) reference manager based on the database and article type. Studies that failed to meet both criteria were removed. Saved references were exported as XML files, and duplicates were removed using Excel Version 2010 (Microsoft, Redmond, WA, USA). A team of three, one author and two trained research assistants, screened articles through a Qualtrics XM (Qualtrics, Provo, UT, USA) survey for criteria of acceptance into both forms of analysis and data collection. Each article was screened twice, once by the author and again by one of two research assistants. The two reviewers for an article discussed disagreements regarding acceptance until they reached a consensus. The requirements for meta-analyses ensured that the following were reported: (1) sample size, (2) source for variance values, and (3) pre-intervention and post-intervention evaluation values. The criteria for acceptance into the thematic synthesis was to ensure the study contained information about one of the following: (1) barriers to online educational programs, (2) feedback or perceptions of the online educational programs, and (3) benefits of training and experience. Additionally, articles were screened to ensure there was no in-person effect on the intervention. The survey also collected numerical data required for the meta-analysis of the studies. Articles for the meta-analysis were evaluated for risk of bias using ROBINS-I and ROB-2 templates based on the study design as required by the tools [23,24]. Articles included for the thematic synthesis were evaluated for quality using a modified critical appraisal skills programme (CASP) checklist [25]. These tools also assisted in collecting data about demographics and study characteristics.

#### 2.1.3. Data Extraction

Detailed quantitative results (i.e., outcome data) for the efficacy of interventions were extracted from each study using the Qualtrics XM survey. Relevant outcome types included dichotomous and continuous measures. Dichotomous measures were noted by the table numbers in which data are located for copying later, while continuous measures were extracted with their associated sample size and variance. When variance was not available, other statistics were extracted (e.g., *p* and *t*-test values) that could be used to estimate an effect size. The missing statistics were calculated with the extracted values using the RevMan Calculator hosted on Cochrane [26].

### 2.2. Random-Effects Meta-Analysis

The data collected were stratified into subgroups by populations of (1) students, (2) food workers, and (3) consumers. Within these subgroups, three main outcome types were considered: (1) knowledge, (2) attitudes, and (3) behaviors. Given that studies used different measurement instruments and scales, a form of the standardized mean difference (SMD) corrected for low sample sizes, Hedge’s G, was selected as the primary effect size metric, as shown in Equation (1).

G = (1 − (3/(4 × n_1_ + n_2_ − 2) − 1)) × (X_bar_1_ − X_bar_2_)/S_p_ (1)

where X_bar_1_ is the mean score of the control group (if no control group exists, the pre-intervention score is substituted), X_bar_2_ is the mean score of the experimental group after treatment, and n_1_ and n_2_ are the sample sizes of the control and experimental groups, respectively. S_p_ is defined in Equation (2) below as:

S_p_ = ((n_1_ − 1) × S_1_^2^ + (n_2_ − 1) × S_2_^2^)/(n_1_ + n_2_ − 2))^(1/2)^ (2)

S_1_ and S_2_ are the sample standard deviation of the scores for the control group and the experimental group, respectively. Hedges G can be interpreted as follows: (1) G = 0.2 as a low effect, (2) G = 0.5 as a moderate effect, and (3) G = 0.8 as a large effect [27].

Studies that reported dichotomous outcomes were converted to Hedge’s G, assuming that all studies measured the same outcome construct. Within meta-analysis subgroups, some studies reported more than one relevant outcome measure. These measures were combined into single values with a fixed-effects model weighted mean, as suggested by Hedges et al. [28].

A random-effects analysis was conducted within groups that contained more than one study to identify variance between studies and calculate an average effect. For calculating the initial estimate for interstudy variance (τ^2^), the method described in Hedges et al. was used [28]. This calculation was refined using the restricted maximum-likelihood estimator (REML) adapted from Sidik and Jonkman, using a convergence criterion defined in Equation (3) [29].

|τ^2^_new_ − τ^2^_old_| < 0.001 (3)

where τ^2^_new_ is the newly calculated difference between study variance and τ^2^_old_ is the previously iteratively calculated difference between study variances. A list of the statistical tests, statistics calculated (e.g., heterogeneity measures), and graphs created for the analysis can be found below in Table 3. All *p*-value tests considered statistical significance at *p* < 0.05. All calculations for the study were performed in Excel using the XRealStats [30] package version 7.6 and self-authored Visual Basic for Applications (VBA) code validated by textbook examples when needed. Finally, a Grading of Recommendations Assessment, Development and Evaluation (GRADE) was conducted for each group to evaluate the overall quality of the groups [21]. An author and another researcher assessed the evidence together to assign scores for the GRADE to obtain a consensus on the evaluation.

### 2.3. Thematic Synthesis

Thematic synthesis of all relevant articles was conducted using the approach described by Thomas and Harden, which aimed to develop “analytical themes” to advise design in future online food-safety educational programs [37]. Thematic synthesis is adaptable for a systematic review and captures first-order and second-order data constructs. The definitions of these orders, along with a third-order construct adapted from Britten et al., can be found in Table 4 [38]. First-order data are generated from one’s own interpretation of a lived experience. Examples of this order include quotes from interviews or surveys. A second-order datum is the interpretation of someone else’s lived experiences. Examples of this order include thematic analysis from interviews and observations of researchers.

The analysis consisted of identifying potentially relevant information, followed by the coding process. One of the authors and two trained research assistants reviewed each study and annotated the construct order of relevant qualitative data. Qualitative data were considered relevant if they met one of the following criteria: (1) identify feedback or opinions about online educational programs as a media, (2) identify insight into online educational programs design that would have been beneficial, and (3) identify potential barriers or issues faced with online educational programs. Identified data were then coded by adapting the process from Chen et al. [41]. Created codes were grouped into deductive themes that could be classified as recommendations or barriers. The analysis was conducted using PDFs of full articles imported into the Nvivo 12 qualitative analysis software Version 12.6.1.970 (QSR International, Doncaster, Australia).

The Confidence in the Evidence from Reviews of Qualitative research (CERQual) approach [19,42] was adapted and used to assess how much confidence to place in each of the findings. CERQual is analogous to the GRADE approach used for the meta-analysis. CERQual evaluates four categories: (1) methodological limitations, (2) coherence, (3) adequacy of the data, and (4) relevance. Based on the assessment of the criteria, the confidence for evidence for a specific subgroup was determined to be one of the four levels: (1) high confidence, (2) moderate confidence, (3) low confidence, and (4) very low confidence.

## 3. Results

### 3.1. Search, Bias, Quality, and GRADE Results

**Figure 1:**
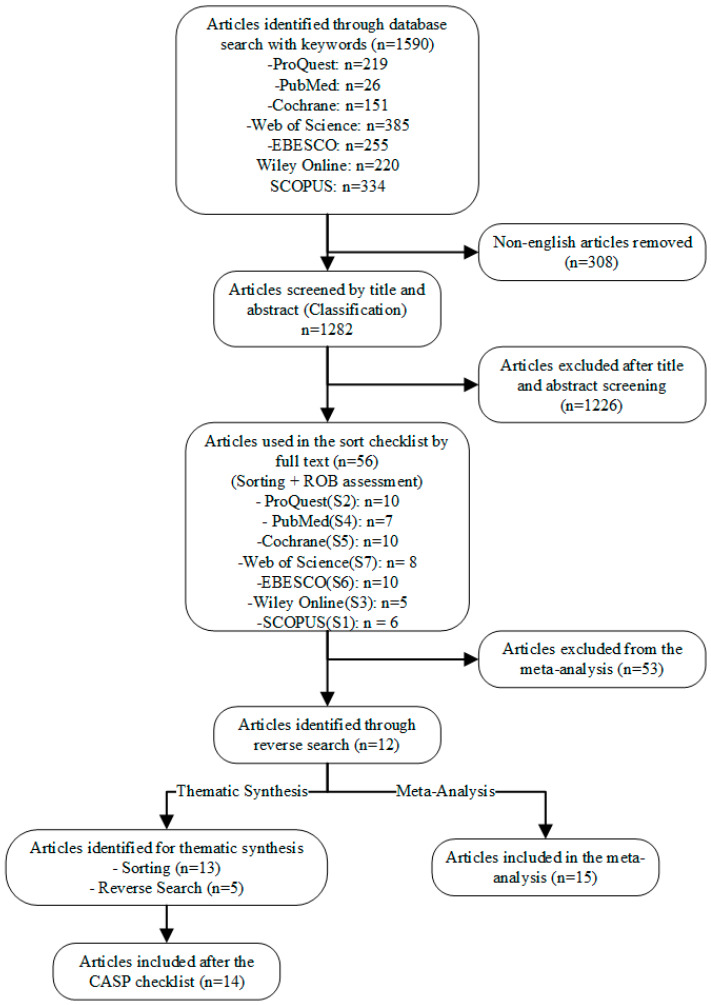
Flowchart showing study selection for the review of the meta-analysis and thematic synthesis.

After sorting and assessing the risk of bias, 22 articles from 1590 search results were identified, 15 for the meta-analysis and 14 for qualitative synthesis, with 7 articles being included in both analyses. A review flowchart of the process is shown in Figure 1. Detailed study characteristics can be seen in Table 5.

The locations of the studies collected were homogeneous. Fourteen (93.33%) of the articles for the meta-analysis were from studies conducted in the United States of America (USA). The last study (6.66%) was conducted in Turkey. Of the 15 studies identified for the meta-analysis, 3 (20%) were RCTs that provided the highest-quality evidence, 7 (46.66%) were pre-post designs, and 4 (26.66%) were NCTs. The kinds of programs included in the meta-analysis varied. Of 15 included studies, 4 (26.66%) are interactive computer programs, 4 (26.66%) are computer modules consisting of various digital media but are not necessarily interactive, 3 (20%) are videos, 2 (13.33%) are web-based programs, 1 (6.66%) is an online course, and 1 (6.66%) is a course delivered via email. Six (40%) of these studies are on the food workers group, five (33.33%) are on the students group, and five (33.33%) are on the consumers group. The study by Barret et al., 2020, is the only study featuring both consumers and students. The study reported separate measures for adults and youths [43]. The youths were assumed to be students, and adults were assumed to be consumers. The studies for the qualitative analysis primarily consisted of studies conducted in the USA. Of the 14 included studies, 10 (71.4%) were conducted in the USA. The remaining studies were conducted in England, Portugal, France, and India. Of the 14 qualitative studies, 3 (21.43%) studies were on consumers, 7 (50%) studies were on food workers, and 4 (28.57%) were on students.

Full risk of bias, GRADE, and CERQual results can be seen in Tables S2 and S3 and Spreadsheets S1, S2, S3, S4. Most studies included in the meta-analysis had a low risk of biases, according to ROBINS-I and ROB-2. Only 5 out of the 15 studies had a moderate or serious risk due to confounding effects. For example, Costello et al. (1997) failed to consider participants’ level of education and ethnicity, which could be potential confounding domains [40]. GRADE and CERQual evaluations were conducted to assess the findings associated with each subpopulation for quality. Low heterogeneity was identified among the studies in each subpopulation (all τ^2^ < 0.1, I^2^ < 20%), suggesting studies are adequately grouped within each category [31]. However, due to the low sample size, many studies consist of NCTs and many consist of self-reported outcomes, and GRADE was adjusted to have low findings. The resulting assessment yielded that most effect sizes ended up with negative values for GRADE or very low confidence. Additionally, much of the qualitative evidence comes from researcher-based, biased second-order constructs. This resulted in lowering confidence in the CERQual assessment.

The CASP checklist was used to check the quality of studies included in the thematic synthesis. A full list of results can be found in Table 6. The results showed that most studies were of moderate quality. The most frequently deficient quality criteria included the following: “Was the research design appropriate to address the aims of our research?” (70% Yes); “Has the relationship between research participants been adequately addressed?” (66.67% Yes); and “Were the qualitative data collected in a way that optimally answers our research questions?” (33% Yes).

### 3.2. Meta-Analysis Results

Information such as effect sizes for individual studies can be found in Figure S1. Publication bias tests indicated no bias, and these tests can be seen in Spreadsheet S5. The knowledge effect size (G = 0.58, *p* < 0.001, n = 12) suggests that online food-safety educational programs have a medium effect. Additionally, the practice effect size (G = 0.42, *p* < 0.0008, n = 5) suggests that online educational programs have a moderate effect on improving food handling practices. However, the attitude effect size (G = 0.29, *p* = 0.078, n = 5) is a statistically non-significant low effect.

### 3.3. Subgroup Meta-Analysis and Thematic Synthesis Results

Based on the meta-analysis results, the effectiveness of online educational programs on each subpopulation is varied, as seen in Table 7. I^2^ is low across groups. However, the confidence intervals for I^2^ values are considerably larger. The meta-analysis results for consumers, students, and food workers, as well as the barriers and recommendations for these subpopulations, are reported in order.

Consumers have a knowledge effect size (G = 0.74) and practices effect size (G = 0.35) that indicate a moderate to possibly high effectiveness and low to possibly moderate effectiveness, respectively. They are the largest group and the most culturally diverse. However, consumers were noted to have experienced (1) technical difficulties and (2) difficulty focusing [51,60]. Recommendations for consumers were limited, and not enough evidence was collected to synthesize results.

Students had a knowledge effect size (G = 0.72) indicating a moderate-to-high effectiveness. Students had both an attitudes effect size (G = 0.23) and a practices effect size (G = 0.30) indicating low effectiveness for both outcomes. Additionally, the knowledge effect size (G = 0.73) has the largest between study variance of all groupings (τ^2^ = 0.094) and is reflected in the confidence interval (CI = 0.3). Three of the studies focused on college-aged students, while two focused on middle- or high-school students (Figure S1). Larger study-level knowledge effect sizes (G = 1.5, G = 0.94, G = 0.55) are associated with older college students, while smaller study-level knowledge effect sizes (G = 0.68, G = 0.34) are associated with younger K-12 students [43,48,53,54,56]. Students struggled with a lack of time, using virtual technology, and social isolation [42,44]. In Fajardo-Lira and Heiss, students reported that the training pace was “moving too quickly” to learn effectively, which resulted in some skipped material [48]. Debacq et al. also noted increased isolation and stress as their students were asked to keep their cameras off in synchronous sessions, that students’ computers would slow during aspects of the course, and internet connection problems were sometimes an issue [44].

Some recommendations identified for students were to (1) increase social interaction and (2) use pop culture, gamification, and videos. Increasing the amount of social interaction in training can be accomplished through incorporating more virtual one-on-one interactions and social media [44]. Debacq et al. recommended increasing the number of interactions between the instructor and students [44]. Mayer and Harrison [54] and Lynch et al. [53] suggested that social media or discussion boards can increase social interaction and create a social learning environment. Additionally, Debacq et al. noted that students appear to enjoy more pop-culture references and games in learning [44]. Mayer and Harrison discovered that students preferred videos as a method of information delivery and that they should be four to seven minutes long [54].

Food workers had a knowledge effect size (G = 0.38) and an attitudes effect size (G = 0.35) that both indicate low to possibly moderate effectiveness. Food workers also were noted to have experienced barriers with (1) turnover, (2) varying educational levels, and (3) a lack of time [50]. Fenton et al. (2006) stated that employees were given only one hour to complete the training [50]. Still, some employees needed more time because they had “difficulty reading the material”.

Some recommendations for food workers that were identified are: (1) use extra resources, (2) use evaluations, and (3) use videos. Temen et al. noted that using extra resources to help those struggling with difficult content was shown to help [59]. Costello et al. recommended that evaluations are necessary to ensure that the content is understood [40]. Regarding findings for students, Temen et al. found that including videos in training generated higher engagement and more improvement in their study [59].

## 4. Discussion

### 4.1. Search Results Indicate Potential Challenges with the Search and Analysis

This review used a structured approach to identify and synthesize available evidence on the effectiveness of online food-safety educational programs in the different subpopulations of students, food workers, and consumers. Additionally, the review employed a thematic synthesis to combine barriers and recommendations for these subpopulations. This review is the first to report an effect size for only-online food-safety educational programs. The types of programs investigated by this study are diverse and grasp an understanding of the effectiveness of online food-safety educational programs. However, most of the studies from the meta-analysis (n = 14) and qualitative synthesis (n = 11) were in the USA. This is a finding similar in many meta-analyses on different topics within food safety education, in which a majority of the studies were found to be conducted in North America ([6,63,64,65,66]). This attribute can have an impact on external validity when extrapolating beyond the USA. The United States is an example of a Western, educated, industrialized, rich, and democratic (WEIRD) society [67]. WEIRD is a large collection of characteristics involving cultural and environmental factors that may not represent aspects of other parts of the world in sociological and psychological characteristics. Cultural factors, such as factors in Hofsted’s cultural taxonomy [68], are demonstrated to affect the way students learn, their learning styles, perceptions, motivational orientation, and achievement, albeit in a potentially minor fashion [69,70,71,72,73]. The effect of cultural factors has also been explored in studies on workplace food safety culture and argued to be considered in studies on behavior change [74,75]. Additionally, sociocultural approaches in adult education emphasize how the social, cultural, and political environments influence adult learning and development [76,77]. It is important to recognize that many of the findings from these studies may not be best extrapolated to other settings without appropriately considering differences in culture and environment underlying these different populations. As such, researchers may want to attempt to investigate effectiveness in other cultures since the nature of online training enables easy interaction with the rest of the world.

The search did not collect non-bibliographic sources (i.e., grey literature or PhD dissertations) or articles in a language other than English due to a lack of resources to enable these inclusions. Other studies may have been published with negative results or were not written in English and may have been missed [78]. Pham et al. conducted a random sample investigation on meta-analysis to explore the effects of limiting the search to bibliographic sources [79]. Random samples of meta-analyses from the agri-food public health area found that up to five articles included in these studies would have been kept out of the analysis. This suggests that some potential articles may have been found if searching through non-bibliographic sources. Additionally, a meta-analysis by Young et al. (2019) [6] on food handler training and educational interventions identified only two non-English articles. However, they noted there might have been more articles in different languages not listed in the bibliographic databases they searched. It is unknown the exact number of articles missed in the search; it is likely that some were missed.

The impact of the study designs in this meta-analysis was reflected in the GRADE and CERQual assessments by the lowering of the confidence in findings. Our search results collected only three RCT trials that provide the highest quality of evidence. With seven collected studies consisting of pre-post uncontrolled designs, some concern should be raised about potential of bias in the results for grouping due to these studies being susceptible to bias [80]. This was reflected in our GRADE assessment as most of the final scores were negative, suggesting low confidence. Pre-post studies are likely more commonly used due to difficulties in recruiting participants [81]. However, this study design suffers from major internal validity issues by assuming the intervention directly causes the outcomes, ignoring uncontrolled factors [82]. Maier-Riehle and Zwingmann identified in their meta-analysis that an effect size for these single-group pre-post designs may likely be overestimated [83]. It is recommended to use a randomized control trial as well as Consolidated Standards of Reporting Trials for Sociological and Psychological Interventions guidelines for designing future studies to limit issues [84]. The CERQUAL assessment on individual studies (Spreadsheet S2) identified that, on average, 94% of codes were based on second-order constructs. Furthermore, the modified CASP questions reveal that only 33% of the included studies conducted their study in a way that is optimal for the analysis. This suggests that results will be based on the researcher interpretation of events without any guarantee for a validified form of data analysis on primary qualitative data, which is a potential source of bias. This was reflected in the downgrade in the CERQUAL assessment.

While most studies were found to be good quality and to have a low to moderate risk of bias, these studies also may be featured in other meta-analyses with different assessments of bias and GRADE results [6,12,22,63,64]. According to Young et al. [6], GRADE requires some judgment to determine appropriate grading criteria and may cause different conclusions based on the researchers. While this study had two reviewers independently assess GRADE and ROBINS-I, this may not be enough to ensure commonality in assessment.

### 4.2. Both Knowledge and Behavior Effect-Sizes Were Moderately Effective

Currently, no other meta-analysis calculates composite effect sizes for food safety educational programs that include different populations together. However, comparisons can be made with existing meta-analysis literature reviews of online food-safety educational programs in other populations (Table 8).

The knowledge effect size was found to be moderately effective, suggesting that online food-safety educational interventions are effective at communicating food safety information. This finding remains consistent with the effect sizes reported in previous meta-analyses [6,12,22,64]. While the knowledge effect size was effective, it is not the only factor that can change behavior [6,85]. Attitudes may play a role in food safety behavior change.

The attitudes effect size was low and found to be statistically insignificant from 0 (G = 0.29, *p* = 0.078), suggesting that the ability of online food-safety educational programs to change how food handlers view food safety is minimal. This value is slightly lower than the attitudes standard mean difference (SMD) effect size calculated in Insfran-Riverola et al. [12] (SMD = 0.29). However, Insfran-Riverola et al. [12] concluded that the effect was moderate despite the calculated effect size being much closer to what is considered low effectiveness based on criteria in Durlak [27] and Borenstein et al. [86]. In contrast, interpretations are cautioned to be relative based on the intervention [87]; larger effect sizes are seen in the results of previous meta-analyses [6,22,64], questioning the interpretation of the findings. Regardless, the effect sizes are similar in calculations, as seen in three other meta-analyses [6,22,63]. Specifically, the results are like the findings in Young et al.’s meta-analysis [6] on food handler food safety training in which they describe a low to moderate effect seen in non-randomized controlled trials on attitudes. Attitudes play an important role in determining food safety behaviors [88,89,90]. Changes in attitude-related variables are important factors that precede behavior change and are included in several models of behavior change [91,92,93]. Following this, it has been shown in several meta-analyses that positive attitude change corresponds with a positive change in behavior [65,94,95]. This suggests that a low attitude effect-size may be a barrier to improving behavior change outcomes in interventions. It is recommended that future studies focus on improving attitudes by stressing the importance of risk and good food safety practices [96].

The behavior effect size was found to be of moderate effectiveness. This is seen in observed behavior effect sizes for Soon et al. [64] and Insfran-Riverola et al. [12]. The effect size of randomized controlled trials for adult educational programs by Young et al. also was of similar effectiveness [63]. However, all other effect sizes from Young et al. were considerably lower [63]. Additionally, Young et al. [6] and Young et al. [22] both calculated a larger effect size for NCTs. The current meta-analysis that calculated the effect size for this study consisted of only a single RCT, two pre-posts, and three NCTs. Considering the study designs by Young et al. [6] and the NCT effect size by Young et al. [22], it is expected that our study’s calculated effect size for practices would be closer to these studies’ effects because the composition for our effect size is mainly NCTs. Our smaller effect size may suggest a difference in effectiveness between online food-safety educational programs and in-person food-safety educational programs. However, more research with a comparative meta-analysis would be required. Despite any potential lower effectiveness, online food-safety educational programs are an effective methodology to improve food safety behaviors by participants. Additionally, many of the behaviors in the studies were self-reported behaviors, which are heavily affected by the instrument used [97]. Furthermore, these measurements are likely to be heavily influenced by social desirability, which may cause overestimation [98,99,100]. However, Young et al. also discuss how some studies have shown agreement between observed and reported behaviors in studies with valid and reliable measurement instruments [63]. It is not evident that the evaluations used in the collected studies have verified the surveys used to measure behaviors, which suggests that the actual behavior effect size may be lower than was calculated. Additionally, a previous meta-analysis separated inspection scores, observed behaviors, and self-reported behaviors into separate categories [6]. That was not considered for this meta-analysis due to the low sample size and low numbers of these studies. For example, Duong et al.’s research was one of the only studies to report observed behaviors. It is unknown what effect combining the measures would have on the effect sizes reported without having correlations assessed [47].

When compared to other meta-analyses on food safety interventions, the results suggest a reoccurring pattern that is seen in both online food-safety educational programs and in-person educational programs. Knowledge change is the highest effect size, with behavior change being the second highest and attitudes being the lowest [6,12,22,63,64]. These patterns are seen with meta-analyses of food-safety educational programs of any method of delivery, suggesting little potential difference between in-person food-safety educational programs and online food-safety educational programs in terms of the relative effectiveness of different outcomes. While this observation may seem to conflict with the conclusion drawn from the behavior effect size, the finding from the pattern relates to the relative order of effectiveness, not the actual magnitude suggested by the behavior effect size.

### 4.3. Subpopulation Analysis

The current meta-analysis explored the effectiveness of online food-safety educational programs and the barriers and recommendations for different populations. The populations explored were consumers, students, and food workers. The implications for some of the results of the meta-analysis and the thematic synthesis are discussed in order of consumers, students, and food workers.

Consumers had a knowledge effect size of G = 0.74, similar to the large effect size of SMD = 0.87 reported by Young et al. for randomized controlled trials with adults [63]. This suggests that online food-safety educational programs are similarly effective at knowledge change in consumers as the overall effectiveness of food safety educational programs of any format to change knowledge in consumers. Consumers also have a low to moderate effect on practices, which is similar to the effect size for non-randomized controlled trials found by Young et al. [63]. This is surprising because the design make-ups of the studies for consumers consist of three pre-post studies and two RCTs. Due to the high amounts of heterogeneity noted in Young et al. [63], it is difficult to determine if the results are similar due to an overestimated bias from the pre-post studies or due to random chance.

Consumers faced (1) difficulties in focusing and (2) technical difficulties. Difficulty in focus is a barrier similar to other noted barriers in adult learning, which is an adjacent topic as consumers are typically adults. Trepka et al. identified that consumers had a difficult time focusing on learning when they had to focus on taking care of their kids [60]. This would be classified as an external barrier that emerges due to personal responsibilities or other influences beyond their control [101,102]. Another traditional type of barrier to adult learning is institutional barriers or barriers constructed by educational bodies, such as researchers, that make participation challenging [103,104]. Technical issues may be considered an institutional barrier as they emerge from the choice of delivery format made by the educational body. Technical difficulties have been linked to lower test scores for adults in online training programs, which can cause attrition in longer-term programs [105]. This suggests that technical issues can pose a large barrier to effectiveness in interventions. Therefore, as technology grows and more tools become available, it is necessary to also put in efforts to assist consumers in learning how to use the technology [104,106]. Technical issues are a barrier unique to online food-safety educational programs. To overcome this barrier, Zirkle and Fletcher claim that having technical assistance in place and maintained is key to any successful online educational program [104].

While no recommendations were identified in the thematic synthesis for consumers, the principles of adult education may be applied when educating consumers. Knowles states that adults are independent and self-directed and need to know the rationale for what they are learning [107]. Knowles et al. outline the principles of andragogy, the art of helping adults learn. The tenets are (1) the learner’s need to know, (2) the self-concept of the learner, (3) prior experience of the learner, (4) readiness to learn, (5) orientation to learning, and (6) motivation to learn [108]. While many of these key assumptions may lack holistic empirical evidence, they can serve as a starting place when designing consumer online food-safety educational programs [109]. While these principles are older, a modern system built upon Bloom’s taxonomy, andragogy, transformational learning, constructivism, and communities of practice, called “virtual andragogy”, has been proposed by Greene and Larsen, who indicated that successful online educational programs for adults will engage the following elements: (1) readiness to learn and understanding, (2) need to know and remembering, (3) experience and applying, (4) orientation to learning and analyzing, (5) self-concept and evaluating, and (6) motivation to learn and creating [110]. However, as a more recent theory, few other publications have been published to validate such a model. These proposed frameworks can operate as design principles when designing online food-safety educational interventions for consumers. While consumers are an important population to educate to assist in reducing foodborne illnesses, the effectiveness, barriers, and recommendations for online food-safety educational programs are different for students.

Students had a large effect size for knowledge and low effect sizes for attitudes and behaviors. This suggests that students are learning about food safety but are often not changing behaviors or views on the importance of food safety. This appears to be the first effect size reported for online food-safety educational interventions for students. Despite this, other meta-analyses have been conducted to look at the effectiveness of online educational programs for students. Ulum conducted a meta-analysis and found a moderate effect size for outcomes in students. However, their subgroup analysis on only studies from the USA reported a low effect size [111]. Weightman et al. found that a meta-analysis on information literacy skills for students was large (G = 0.92) [112]. These previous meta-analyses support the findings that online food-safety education is an effective way to deliver food safety information to students. Finding a good comparison is difficult, as many studies instead focus on comparative effectiveness between in-person and online educational programs and typically only measure knowledge [13,113,114].

Results also identified that larger effect sizes are more associated with college-level students (n = 3), while smaller effect sizes are affiliated with younger K-12 students (n = 2). This suggests that perhaps online educational interventions may be more effective for older students. While the sample size may be small, the differences in magnitude suggest a possible hypothesis backed by other evidence and theory. This result was similar to a meta-analysis finding by Means et al. on online educational programs [13]. Turan et al. identify in the beginning of their literature review that traits for success in online education are (1) self-regulation, (2) satisfaction, (3) perceived flexibility, and (4) independence [115]. Turan et al. find that literature suggests that self-regulation and perceived flexibility were noted to predict satisfaction, which was highly associated with (1) dropout rate, (2) motivation, (3) determination to complete a course, and (4) success rates [115]. Self-regulation is slowly developed from childhood to adulthood, albeit with some level of heterogeneity [116,117]. This suggests that these are traits mostly possessed by adults and may explain why online educational interventions are more effective for older students. However, Barbour and Reeves identified that most of the younger students enrolled in an online school were more successful when they had a higher level of self-regulation [118]. While more evidence is needed to confirm if online food-safety educational programs are more effective in older students than younger students, it is clear that it is also important to teach self-regulation to younger students for them to be successful.

One of the barriers identified for students was a loss of social interaction. Hermanto and Srimulyai identified this barrier in their study of students participating in an online educational program during COVID-19 [119]. Social interaction is an extremely important aspect of education, especially for adult learners, such as college students [120,121]. It plays a huge role in personal motivation for learning [122]. Additionally, social interaction can compound in a negative effect with other barriers [123]. For example, the current study identified that the loss of social interaction was noted with increased stress. The exact effect of stress varies from person to person, but in some cases, it can affect learning outcomes [124]. Stress can also negatively impact learners’ attitudes toward what is being taught, but the overall effect is not fully understood [124]. Furthermore, stress and difficulty focusing suggest an underlying lack of motivation to complete the training [120,125]. Training needs to be interactive and rewarding to be motivating and effective, especially for college students [120,125,126]. Overall, this barrier can present a huge problem by potentially limiting the effectiveness of the training. Ivanec noted in a survey of Croatian university students that those who perceived greater social isolation also noted greater difficulties with learning and self-regulation in studying [127]. As such, future studies should identify a means to implement more social interaction in their online food-safety educational programs.

One recommendation for students that may assist with generating more social interaction is to use more videos because they tend to generate engagement. Furthermore, Dailey-Hebert also suggested that videos can improve interpersonal student–teacher relations while helping students to learn [128]. In addition to recommendations to students, the current analysis also suggested further investigating the efficacy of food safety education programs for food workers.

Food workers have a low to moderate effect size in knowledge (G = 0.38). This is lower than effect sizes from findings in meta-analyses on food handlers, for which knowledge was found to have a large effect size [6,12,22,64]. This suggests that when comparing online food-safety educational programs to in-person food-safety educational programs, regardless of their instructional format, these programs may be below average in effectiveness at improving food safety knowledge in food workers. However, a more rigorous investigation is warranted as the current meta-analysis was not designed as a comparative analysis. Furthermore, this small effect size may be due to a study by Feinstein et al. that was initially kept in our meta-analysis results but was removed as it was an outlier with a very large effect size [49]. This will be discussed later in the limitations. Despite the effect size in the population being lower than the other studies, the statistically significant effect suggests that online food-safety education is effective at increasing knowledge for food workers, albeit perhaps to a limited degree.

One of the barriers noted in this group is the high turnover rate, which lowers peer collaboration and communication [129]. Cornelissen found that peer collaboration and communication support knowledge spillover that supports learning [130]. Additionally, turnover may reduce employee motivational investment in training. Royalty suggests that human capital theory predicts that workers will be more likely to invest in job training the longer they expect to remain working [131]. They found that the likelihood of training success can be attributed to differences in turnover by education level rather than just a pure interaction between education and training. Overall, high turnover has a negative impact on employee training.

Another noted barrier was varying education levels, which can cause issues in understanding the educational content. Fenton et al. recommended looking at the design of the training for appropriate difficulty, especially for reading level [50]. One method of implementing this would be to apply adapting learning paths. Such a form of personalized training is becoming more popular due to newer technologies that can adapt and modify things as feedback is received [132]. Multiple studies have shown that automated adapted learning paths benefit various populations [133,134,135]. However, no study has yet evaluated the effectiveness of such programs for food workers and may present a potential opportunity to improve online food-safety educational programs for food workers.

One other barrier noted in the population was a lack of time for training. This was a barrier also identified in other studies on different food handlers and managers [136,137,138]. With the prevalence of the barrier, training that takes less time may help to alleviate these barriers [139]. Sandlin summarized the literature and identified that online educational programs have been identified to be more time- and cost-efficient than in-person alternatives when it comes to implementations [140]. Online educational programs are also flexible [141]. It may be best to design online educational programs that are shorter in duration for food workers than those currently implemented, which has been demonstrated to be possible without decreasing their effectiveness [142,143].

### 4.4. Limitations

Despite the carefully developed protocol, some limitations remain that need to be acknowledged due to assumptions made in the methodology as a compromise to include more studies in the analysis. Other limitations emerged from the collected studies in the study.

To accommodate for more studies, *p*-values were used to estimate variance. However, exact *p*-values were not reported in some studies. These reported *p*-values were used as if they were the actual *p*-values, which would conservatively overestimate the variance. Additionally, while none of the publication bias tests reported the existence of publication bias, it is still a concern in any meta-analysis.

Several limitations were found to be due to the nature of the collected studies. Many studies failed to meet the inclusion criteria for the meta-analysis because of missing required metrics, confounding interventions, and failure to collect a pre-intervention score [44,51,55,59,144,145,146,147,148,149]. Therefore, not all available or collected data could be synthesized, which is the primary goal of a meta-analysis. Additionally, a lack of available studies reflects the small number of studies that can be included in subpopulation analysis. One standard method used to evaluate outcomes in the collected studies is evaluation using tests. These tests can suffer from a ceiling effect when calculating effect size and regression to the mean (RTM) biases [82]. The ceiling effect occurs when comparing pre-tests and post-tests. If the pre-test mean is high, it limits the magnitude of change that can be measured in the post-test. The RTM occurs and suggests that extremely low or extremely high values tend towards the mean. For example, if a participant scores 90% on a pre-test but scores 85% on the post-test, this decrease is unlikely due to “unlearning,” the idea that the intervention worsened their knowledge, but is instead because of RTM. Future studies are recommended to consider these biases and make efforts to correct or control them, as Barnett et al. suggested [150]. One example that may suggest the existence of the ceiling effect in some studies is Feinstein et al. (2013), which was initially included in the food workers group but had a considerable effect size (G = 2.22) outside three standard deviations from the mean [49]. The inclusion of this study would have increased the food workers’ knowledge effect size to G = 0.78, closer to the effect sizes seen in other meta-analyses, but it was removed due to it being an outlier. Feinstein et al. removed from their reporting all participants who scored greater than 80% on the pre-test, while three out of five studies in the food workers group had pre-score means greater than 80% [49]. This removal of participants would explain why the calculated effect size for the study was so large compared to others in the group, as it would reduce the impact of the ceiling effect. However, it is worth considering if this is an approach future studies should mimic as researchers should identify if the main objective of the educational program is to measure effectiveness in all involved or effectiveness on participants who may need the knowledge.

## 5. Conclusions

Overall, our results present a rough estimate of the success of food safety training for different groups but suggests that online educational programs are effective at improving knowledge in almost all subpopulations. However, there is room for improvement in practices and attitudes. The results may suggest that online food-safety educational programs may be more effective in older students than younger students. However, more research is needed to confirm these findings. The findings from the meta-analysis also need to be interpreted with some caution due to the low number of RCTs and NCTs, as results may be biased. Online food-safety educational programs for food workers are limited in effectiveness due to a lack of time to conduct training, varying educational levels, and high worker turnover. It may be beneficial to design shorter trainings and experiment with adaptive learning paths in training programs. Students struggle with technical difficulties and a lack of social interactions. Students can be more successful with the teaching of self-regulation and building confidence in using technology. Furthermore, including more opportunities for social interactions, such as social media or message forms in the program, may assist with reducing the impact of the loss of social interaction. Training courses should be shorter and incorporate more breaks to improve the experience. Additionally, designing the research to be more interactive with activities can bring benefits, such as improved results in training. To improve the results of future studies, scientists should carefully consider how they measure the study outcomes and what the main objectives of their intervention should be. Ultimately, the end goal of training is to improve practices.

## Figures and Tables

**Table 1:** Inclusion criteria and exclusion criteria based on PICO ^1^ framework.

Inclusion		Exclusion
Language	English	A PhD thesis
P: Population	Is the population a type of food handler?	An MS thesis
I: Intervention	Does the study include an online educational program? Was the focus of the study a food safety topic? (e.g., hygiene, regulations, practices, allergies, quality, etc.)	A review article (systematic reviews or meta-analyses) Only an abstract
C: Comparison	Does the study include a control group or pre-training measure for the experimental group? (quasi-experimental, RCT, non-randomized trials)	Studies with potential confuscation of an in-person training on the online educational program’s evaluation
O: Outcome	Does the study include one or more of the following outcomes: knowledge, practices, or behaviors (KAP)?	

^1^ PICO is a common project design method for comparative studies and is endorsed by Cochrane for use in systematic reviews [20,21].

**Table 2:** Searching keywords used for systematic review.

PICO	Keyword
Population	Industry OR Handler OR Worker OR Employee OR Processor OR Manufacturer OR Farm OR Restaurant OR Retail OR Business
	AND
Intervention	Workshop OR Intervention OR Instruction OR Education OR Curriculum OR Course OR Training OR Brochure OR Strategy OR Lesson
	AND
Digital Specifier	Webinar OR Digital OR Virtual OR Online OR Internet
	AND
Outcome	Practices OR Behavior OR Perception OR Attitude OR Knowledge
	AND
Topic	Safety OR Handling OR Preparation OR Hygiene OR Quality OR Technology OR Allergy
	AND
	Food

**Table 3:** Statistics, metrics, and plots calculated.

Purpose	Statistics	Tests	Plots	Method Adapted From
Effect Sizes	Hedge’s G	95% CI, 95% PI	Forest Plots	Average Effect [28], Intervals [20]
Between-Study Variance	τ^2^	*p*-value	Funnel Plot	Initial [28] REML [29]
Observed Heterogeneity	I^2^	95% CI	N/A	I^2^ [31,32,33] Interval [34]
Publication Bias	Begg’s Ranking	*p*-value	Funnel Plot	Begg’s Ranking and *p*-value [35] Funnel Plot [36]

**Table 4:** Definitions and examples for the different orders of qualitative data constructs.

Order of Construct	Adopted Definition	Typical Forms	Example	Citation of Example
1st level	Direct life experience of evidence. These are typically quotes from people	Quotes	“We don’t have the resources in place at this time to take part in the programme”	[39]
2nd level	Interpretations of 1st-level data generalizations involving an interpretation of other’s life experiences	Scientific observations, codes, or summaries	In terms of practical application, the use of the computer training provided a much easier method to implement	[40]
3rd level	Top-level synthesis of 1st-level and 2nd-level interpretations	Themes from a synthesis	Themes from the result of this study	N/A

**Table 5:** Meta-analysis and thematic synthesis study characteristics.

Study	In Thematic Synthesis	In Meta-Analysis	Population	Digital Method	Type of Design	Outcomes ^†^	N	Country of Focus
[43]	No qualitative data found	☑	Students, Consumers	Video	Pre-Post	KAP	Consumers = 581 Students = 349	USA
[40]	☑	☑	Food Workers	Interactive Computer Program	NCT **	K	Control = 15 Experimental = 14	USA
[44]	☑	No clear variance	Students	Virtual Course (Semi-Synchronous, Interactive)	Pre-Post	K	n = 30	France
[45]	☑	☑	Food Workers	Interactive Computer Program	NCT **	P	Control = 20 Experimental = 20	USA
[46]	☑	In-person component in intervention §	Food Workers	Video	Pre-Post	KP	n = 270	India
[47]	☑	☑	Consumers	Video	RCT	P	Control = 210 Experimental = 182	USA
[39]	☑	In-person component in intervention §	Food Workers	Digital Quiz	Pre-Post	A	n = 11 Businesses	England
[48]	☑	☑	Students	Web-based Program	Pre-Post *	K	Pre-Test = 19 Experimental = 19	USA
[49]	No qualitative data found	☑	Food Workers	Web-based Program	Pre-Post	K	Pre-Test = 343 Experimental = 343	USA
[50]	☑	☑	Food Workers	Computer Module (Recorded PowerPoints with Video Clips)	RCT	KA	Control = 28 Experimental = 35	USA
[51]	☑	In-person component in intervention §	Consumers	Informational Website	RCT	AP	n = 446	USA
[52]	No qualitative data found	☑	Consumers	Online Course	Pre-Post	K	Pre-Test = 76 Experimental = 76	USA
[53]	☑	☑	Students	Interactive Computer Program	Pre-Post	K	Pre-Test = 217 Experimental = 217	USA
[54]	☑	☑	Students	Computer Module	NCT	KAP	Control = 93 Experimental = 278	USA
[55]	☑	No clear variance	Food Workers	Videos	Pre-Post	K	n = 146	Portugal
[56]	No qualitative data found	☑	Students	Digital Game/Interactive Computer Program	NCT **	KAP	Control = 365 Experimental = 903	USA
[57]	No qualitative data found	☑	Food Workers	Video	Pre-Post	K	Pre-Test = 240 Experimental = 240	USA
[58]	No qualitative data found	☑	Food Workers	Computer Module (Recorded PowerPoints with Video Clips, Digital Documents)	Pre-Post	KA	Pre-test = 40 Experimental = 20	USA ***
[59]	☑	In-person component in intervention §	Food Workers	Web-based Program	N/A ▪ (Post Only)	K	n = 21	USA
[60]	☑	No outcomes measured	Consumers	Computer Module	N/A ▪	N/A ▪	n = 180	USA
[61]	No qualitative data found	☑	Consumers	E-mail Course	Pre-Post	P	Pre-test = 34 Experimental = 34	Turkey
[62]	No qualitative data found	☑	Consumers	Computer Modules	RCT	K	Control = 10 Experimental =10	USA ***

* Design was RCT in assignment between in-person and digital, but effect-size is calculated from a pre-post test design. ** Cluster of participants was randomly assigned to the computer or the face-to-face group. Participants themselves were not randomly assigned. *** Assumed based on the country of the publishing author. ^†^ K–Knowledge, A—Attitudes, P—Practices. ▪ N/A—Information is not available as it was not reported or unclear. § Program contained an in-person or non-online aspect that may have affected the numerical results and was not included in the analysis. Qualitative data were checked to ensure that the context was around the online food-safety educational program aspect of the study.

**Table 6:** Modified CASP checklist results.

Question	% No (n)	% Cannot Tell/Partially (n)	% Yes (n)
Was there a clear statement for the aims of the research? (CASP)	0%	0%	100% (30)
Is the methodology of the study appropriate for the topic of interest? (CASP)	3.33% (1)	6.66% (2)	90% (27)
Was the research design appropriate to address the aims of the research? (CASP)	3.33% (1)	3.33% (1)	93.33% (28)
Was the research design appropriate to address the aims of our research?	30% (9)	0%	70% (21)
Was the recruitment strategy appropriate to the aims of the research? (CASP)	6.66% (2)	3.33% (1)	90% (27)
Were the qualitative data collected in a way that optimally answers our research questions?	33.33% (10)	33.33% (10)	33.33% (10)
Were the data collected in a way that addresses the research issue? (CASP)	0%	0%	100% (30)
Has the relationship between research participants been adequately addressed? (CASP)	33.33% (10)	0%	66.67% (20)
Have ethical issues been taken into consideration? (CASP)	3.33% (1)	10% (3)	86.66% (26)
Was the data analysis sufficiently rigorous? (CASP)	6.66% (2)	6.66% (2)	86.66% (26)
Is there a clear statement of findings (CASP)	0%	3.33% (1)	96.66% (29)

n = 30, as these are the findings together by two reviewers on 15 papers. (CASP)—Indicates the criteria was from the original CASP checklist.

**Table 7:** Effect sizes between each subpopulation.

Group	Outcome	Avg. Hedge’s G ± CI 95%	τ^2^	I^2^ ± CI 95%
Consumers	Knowledge (n = 3)	0.74 ± 0.11 **	0 *	0
	Attitudes (n = 0)	N/A ^§^	N/A ^§^	N/A ^§^
	Practices (n = 3)	0.35 ± 0.28 *	0.0459	0 ± 83.62
Food Workers	Knowledge (n = 5)	0.38 ± 0.16 *	0 *	0
	Attitudes (n = 2)	0.35 ± 0.26 * Г	0 *	0
	Practices (n = 0)	N/A ^§^	N/A ^§^	N/A ^§^
Students	Knowledge (n = 4)	0.72 ± 0.30 **	0.094307	18.67 ± 40.67
	Attitudes (n = 3)	0.23 ± 0.28	0.05508	2.75 ± 13.83
	Practices (n = 3)	0.30 ± 0.32	0.07344	4.49 ± 20.32
All	Knowledge (n = 12)	0.58 ± 0.19 *	0.048676	15.07 ± 35.63
	Attitudes (n = 5)	0.29 ± 0.26	0.05382	0
	Practices (n = 6)	0.42 ± 0.22 **	0.043314	0

* Two-tailed *p*-value ≤ 0.05. ** Two-tailed *p*-value ≤ 0.01. Г—Subgroup contains only 2 studies. Variance and dispersion arise in random-effects models when dealing with a small number of studies [33]. ^§^—N/A, as there were no studies in the subpopulation reporting the outcome.

**Table 8:** Characteristics of meta-analysis featuring mainly the population of the United States, considering both online and in-person studies.

Citation	Population	Effect Size Used	Knowledge	Attitudes	Practices
Young et al., 2015 [63]	Consumers	SMD	RCT *Training* Adults = 0.87 *Media Campaigns* Adults = 0.42 NRT Adults = 0.44 Children = 0.24	NRT *Training* Adults = 0.26 RCT *Media Campaign* Adults = 0.34	RCT *Education* Adults = 0.68 Children = 0.2 *Media Campaign* Adult intents = 0.36 Adult = 0.24 NRT *Education* Adults = 0.37 Children = 0.33
Insfran-Rivarola et al., 2020 [12]	Food Handlers	SMD	1.24	0.28	Observed = 0.45 Self-reported = 0.8
Young et al., 2020 * [22]	Food Handlers	SMD	1.104	0.433	0.898
Young et al., 2019 [6]	Food Handlers	SMD	RCT 0.97 NRT 1.77	RCT 0.12 NRT 0.38	RCT 0.18 NRT 1.16
Soon et al., 2012 [64]	Food Handlers	Hedge’s G	1.284	0.683	0.718 **

* Study only includes single group pre-post designs. ** Study labels figures as hand hygiene attitudes, but study characteristics show studies for the chart measure behavioral outcomes.

## Data Availability

The original contributions presented in the study are included in the article/Supplementary Material, further inquiries can be directed to the corresponding author.

## Supplementary Materials

The following supporting information can be downloaded at: https://www.mdpi.com/article/10.3390/foods13050794/s1, Document S1: PRISMA checklist, Table S1: Date databases accessed, Table S2: ROBINS-I assessment summary for studies in the meta-analysis, Table S3: ROB-2 assessment summary results for studies in the meta-analysis, Spreadsheet S1: GRADE-CERQUAL grouping assessment results, Spreadsheet S2: GRADE-CERQual individual studies results, Spreadsheet S3: GRADE grouping assessment, Spreadsheet S4: GRADE individual studies assessment, Spreadsheet S5: Publication bias tests and funnel plots, Figure S1: Study-level individual effect sizes.
