# Supplementary material for: Effectiveness of Online Food-Safety Educational Programs: A Systematic Review, Random-Effects Meta-Analysis, and Thematic Synthesis
Source: Foods. 2024 Mar 4;13(5):794. doi: 10.3390/foods13050794 (PMC10931379; doi:10.3390/foods13050794)
Supplement: Supplementary file 1 [file foods-13-00794-s001.zip › Supplementary Tables and Figures.pdf]

Supplementary Material

Table S1: Date Databases Accessed

Database	Last Access Date
Proquest	August 25th, 2021
PubMed	August 27th, 2021
Cochrane	August 27th, 2021
Web of Science	August 30th, 2021
Wiley Online	August 24th, 2021
EBESCO	August 30th, 2021
SCOPUS	August 16th, 2021

Table S2: ROBINS-I assessment summary for studies in the meta-analysis

Paper	D1	D2	D3	D4	D5	D6	D7	Overall
Barrett et Al., 2020	+	+	+	+	+	+	+	+
Costello et Al., 1997	X	+	+	+	+	+	+	X
Dipietro, 2006	−	+	+	+	+	+	+	+
Feinstein et Al., 2013	+	X	+	+	−	+	+	−
Liceaga et Al., 2014	+	+	+	+	+	+	+	+
Lynch et Al., 2008	−	+	+	+	+	+	+	+
Quick et Al., 2013	+	+	+	+	+	+	+	+
Smith and Shillam, 2000	X	+	+	+	−	+	+	X
Strohbehn et Al., 2013	−	−	X	+	+	+	+	X
Unusan, 2007	+	+	+	+	+	+	+	+

+ = Low risk of bias<br>− = Moderate risk of bias<br>X = Serious risk of bias	D1- Bias due to confounding<br>D2- Bias in participant selection<br>D3- Bias in the classification of interventions<br>D4- Bias due to deviation from the intervention<br>D5- Bias due to missing data<br>D6- Bias in the measurement of the outcomes<br>D7- Bias in the selection of reported results

Table S3: ROB-2 assessment summary results for studies in the meta-analysis

**Paper**	**D1**	**D2**	**D3**	**D4**	**D5**	**Overall**
Duong et Al., 2020	−	+	+	−	+	−
Fajardo-Lira et Al., 2006	+	+	+	+	+	+
Fenton et Al., 2006	+	+	+	+	+	+
Mayer and Harrison, 2012	+	+	+	+	+	+
Walker et Al., 2006	+	+	+	+	+	+

+ = Low Bias<br>− = Some Concerns of Bias	D1- Bias in the randomization process<br>D2- Bias due to deviations from procedure<br>D3- Bias due to missing data<br>D4- Bias due measurement of outcomes<br>D5- Bias due to selective reporting

Figure S1: Study-level individual effect sizes

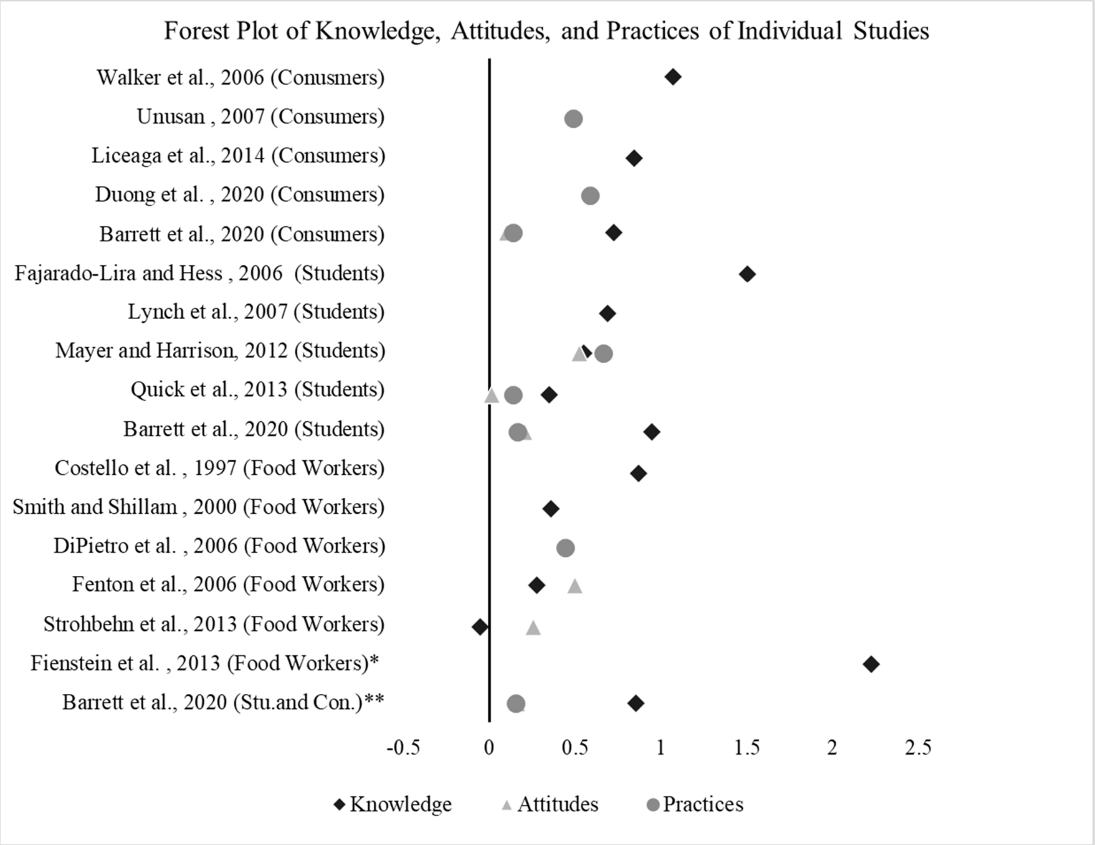

Figure S1: Hedge's G effect size for Knowledge, Attitudes and Practices of each study

Any missing effect size is due to the study not reporting on that outcome.

*** Combined value of Students and Consumers from study using a fixed effect weighed hedge's G*

**Removed from the calculation of the subpopulation's effect size*
